# Notch Signalling Under Maternal-to-Zygotic Transition

**DOI:** 10.1080/19336934.2022.2139981

**Published:** 2022-11-08

**Authors:** Tomoko Yamakawa, Elzava Yuslimatin Mujizah, Kenji Matsuno

**Affiliations:** Department of Biological Sciences, Graduate School of Science, Osaka University, Osaka, Japan

**Keywords:** Notch signaling, maternal effect, maternal-to-zygotic transition, *pecanex*, *almondex*, early embryogenesis

## Abstract

The development of all animal embryos is initially directed by the gene products supplied by their mothers. With the progression of embryogenesis, the embryo's genome is activated to command subsequent developments. This transition, which has been studied in many model animals, is referred to as the Maternal-to-Zygotic Transition (MZT). In many organisms, including flies, nematodes, and sea urchins, genes involved in Notch signaling are extensively influenced by the MZT. This signaling pathway is highly conserved across metazoans; moreover, it regulates various developmental processes. Notch signaling defects are commonly associated with various human diseases. The maternal contribution of its factors was first discovered in flies. Subsequently, several genes were identified from mutant embryos with a phenotype similar to *Notch* mutants only upon the removal of the maternal contributions. Studies on these maternal genes have revealed various novel steps in the cascade of Notch signal transduction. Among these genes, *pecanex* and *almondex* have been functionally characterized in recent studies. Therefore, in this review, we will focus on the roles of these two maternal genes in Notch signaling and discuss future research directions on its maternal function.

## Introduction

Cell signaling plays a pivotal role in the development and homoeostasis of multicellular organisms. During recent decades, molecular mechanisms underlying various cell signaling pathways, such as the Notch signaling pathway discussed in this review, have been extensively studied. Because the components of these cell signaling pathways are mostly encoded in genomes, genetics has been a powerful approach for identifying and characterizing them. The phenotypes observed upon the mutation of these genes have been particularly informative, allowing researchers to dissect the cascades of these cell signaling pathways.

Genetic trait inheritance is commonly governed by Mendel’s laws. However, in some cases, these traits are solely determined by the mother’s genotype regardless of the offspring’s genome. Such maternal effects were first identified in studies on genes that control development. For example, the coiling polarity of the pond snail *Lymnaea* is determined by maternal effects [1,2]. The shells of these snails show dextral (right-handed coiling) or sinistral (left-handed coiling) chirality; dextral and sinistral mutants are dominant and recessive, respectively [1,2]. The shell’s coiling direction is solely determined by the mother’s genotype [1,2]. For example, a mother snail homozygous for the sinistral gene always bears sinistral offspring regardless of her mate’s genotype (or the offspring accordingly). This observation indicates that the coiling polarity of the shell of *Lymnaea* is determined by maternal effects.

In *Drosophila’s* early embryogenesis, the roles of maternal gene functions have been understood. For example, a fly embryo’s anterior-posterior polarity is initially defined by the concentration gradient of a transcription factor called Bicoid, whose concentration descends from the embryo’s anterior end towards the posterior end. The Bicoid protein in the egg is produced from the *bicoid* mRNA provided by the mother. Hence, in the *bicoid* mutant offspring derived from homozygous mutant mothers, both the anterior and posterior ends develop as tails due to a failure in the anterior identity specification [3].

The events of early embryogenesis in flies, including polarity formation, often depend on the maternal functions of genes because these events occur before the activation of the zygotic genes. Early embryos are equipped with mRNAs supplied to the egg by the mother during oogenesis. These maternal mRNAs gradually degrade as embryonic development proceeds. More importantly, various cell signaling pathways play crucial roles in early embryogenesis when maternal mRNAs or proteins predominantly function. Therefore, such maternal functions of genes are closely related to various cell signaling pathways responsible for early embryogenesis. However, it is time-consuming to remove each gene’s maternal contribution to study the corresponding maternal effect, even when using genetic model animals such as *Drosophila*. Thus, the maternal functions of various genes involved in cell signaling pathways are still elusive. Nevertheless, the persistent efforts of many laboratories have revealed the essential roles of some of such maternal genes in cell signaling pathways required for early embryogenesis [4–8]. This review describes the maternal functions of the genes that contribute to early embryogenesis and discusses the recent findings on the roles of maternal genes in the Notch signaling pathway.

### Maternal-to-Zygotic transition

**Figure 1.**
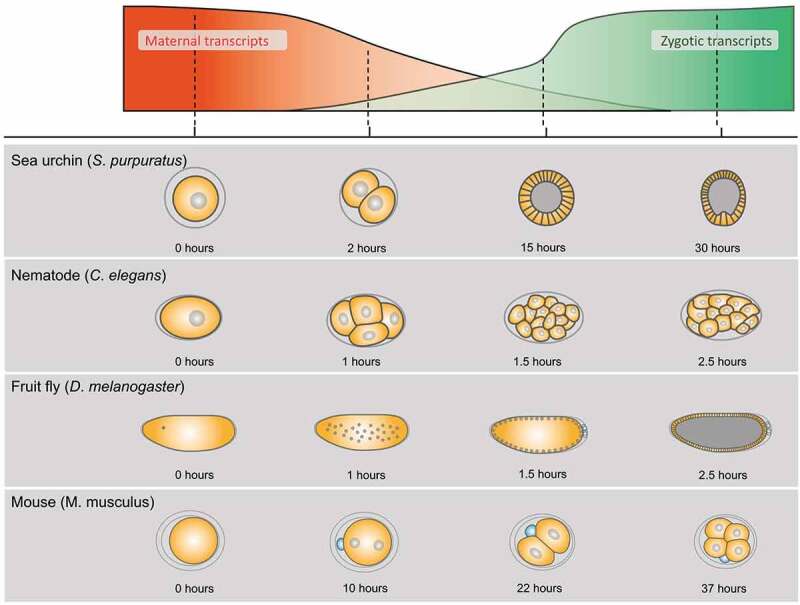
Clearance of Maternal Transcripts and Production of Zygotic Transcripts during the MZT in Various Animals. Schematic diagrams of the MZT in model organisms, including the sea urchin (*S. purpuratus*), nematode (*C. elegans*), fruit fly (*D. melanogaster*), and mouse (*M. musculus*). The maternal transcripts (orange curve) gradually degraded in proportion to the increase of zygotic transcripts (green curve). Each phase's length and the MZT's timing differ among species. The embryonic stages (schemas and hours after the development’s commencement) of each animal are shown, corresponding to the MZT’s phases.

Generally speaking, the embryogenesis of animals is initially directed by maternal gene products. In *Drosophila*, we know that approximately 55–65% of genes show a maternal contribution [9–11]. The functions and activities of maternal mRNAs are post-transcriptionally regulated through multiple mechanism layers. For example, the specific localization, translation efficiency, and degradation of maternal mRNAs are controlled by cis-acting elements within the maternal mRNAs and factors binding to them. Upon the depletion of maternal mRNAs, the transcripts from the zygotic genome start coordinating the embryonic development. Moreover, the ratio of maternal-to-zygotic mRNA amount is reversed during the early embryogenesis of various organisms (Figure 1). This phenomenon is generally called the Maternal-to-Zygotic Transition (MZT) [12]. MZT and its underlying mechanisms are commonly observed among metazoans [13–16]. However, there is a difference in MZT timing among species because the half-lives of maternal mRNAs and the initiation of zygotic transcription differ among organisms (Figure 1). Hence, it is generally important to understand how zygotic genes, which are initially silent, are activated during the MZT. This activation is designated as Zygotic Genome Activation (ZGA).

*Drosophila’s* early embryo is a syncytial blastoderm where nuclear divisions synchronously take place; these divisions are called Nuclear Cycles (NCs) (Figure 1). Early nuclear cycles that comprise the first hour of embryogenesis (NCs 1–8) take only 8 min each, which is too short to allow any zygotic transcription [17]. The gradual prolongation of NCs to 20 min by NC 13 triggers the low level of ZGA. In NC 14, the ZGA’s bulk occurs, initiating the embryo’s cellularization, and lasts for at least 70 min depending on the cell types; this is because cell divisions are no longer synchronous [18]. Simultaneously, a large part of the maternal RNA starts degrading. This is because maternal RNA degradation needs zygotic factors, like miRNAs, even though there is an earlier ongoing RNA degradation by a maternal pathway as well [19]. Recently, the dynamic transition of mRNA species coupled with the MZT has been revealed by genome-wide analyses [20–22].

*Drosophila zelda* (*zld*), which encodes a maternal transcription factor, was identified as a master regulator of ZGA [23]. The translation of maternally deposited *zld* mRNA is induced soon after fertilization and peaks upon the ZGA’s occurrence [23–25]. Zld binds to a class of DNA-sequence motifs that are enriched in the cis-regulatory elements of genes activated in ZGA [26]. The enhancer regions of early expressed zygotic genes have an intrinsically high nucleosome barrier, and Zld overcomes it via the local depletion of nucleosomes [27,28]. Consequently, Zld establishes or maintains regions of open chromatin, thereby potentiating the binding of other transcription factors that promote the MZT. In concert with other transcription and chromosomal factors, Zld establishes a three-dimensional, high-order conformation that assures the expression of zygotic genes.

In this review, we discuss the components of maternally provided Notch signaling that have been extensively studied in *Drosophila*. Since Notch signaling acts through direct cell-cell interactions, the initiation of its functions needs to be coupled with the cellularization of the syncytial blastoderm (Figure 1). This idea is generally applicable to other cell signaling pathways mediated by extracellular signaling molecules and membrane receptors. The cellularization proceeds during the MZT, and a cellular blastoderm is formed in embryos around 2.5 hours post fertilization when ZGA is ongoing (Figure 1) [29]. Thus, shortly after the MZT, various cell signaling pathways activate the zygotic expression of their target genes. Considering the timing of such target-gene activation, we can infer that the components of these cell signaling pathways should be prepared before the MZT. *Drosophila* Notch signaling is no exception because it regulates the differentiation of mesectoderm, which occurs immediately after the MZT [30,31]. Studies have shown that various genes composing the Notch pathway have a maternal contribution that supports early embryogenesis. However, how these maternal genes execute their functions in the Notch signaling during and after MZT remains unclear.

### The Notch signaling pathway

**Figure 2.**
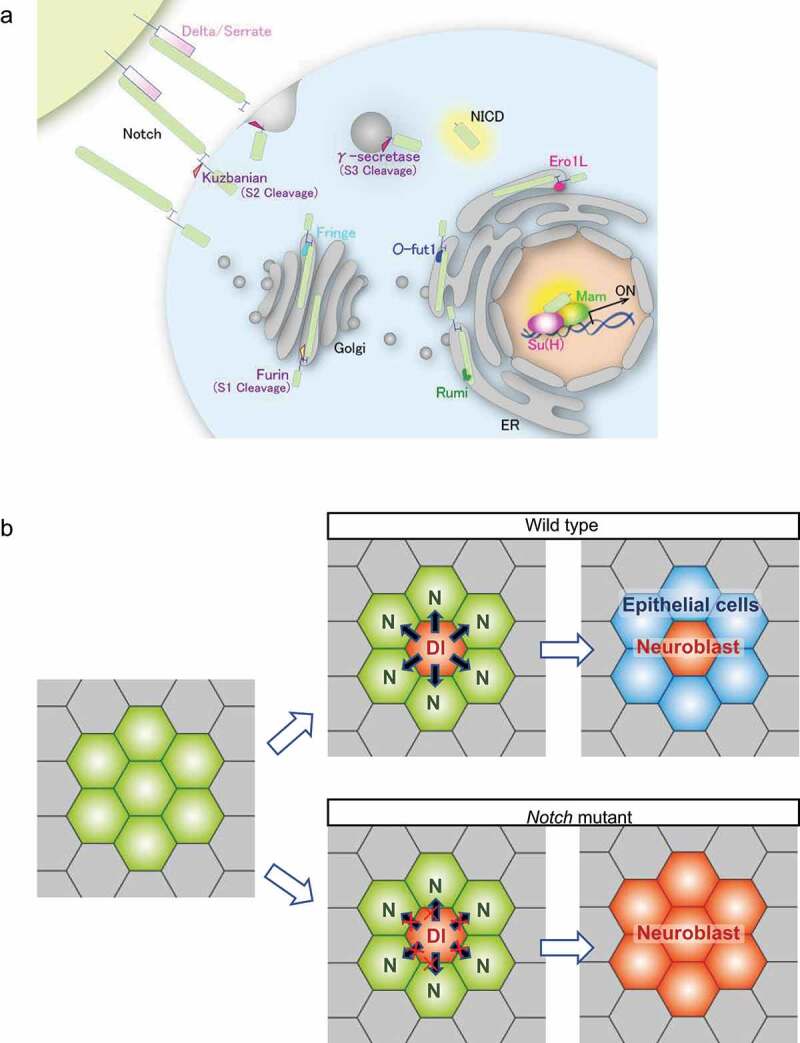
The Notch Signaling Pathway and its Function in Lateral Inhibition. (a) The Notch receptor (green) is produced in the Endoplasmic Reticulum (ER) and matured during transportation from the ER to the Golgi. The Notch is processed by a furin-like protease within the Golgi (S1 cleavage) and then migrates to the cell's surface. Additionally, in the ER and the Golgi, the Notch is glycosylated by *O*-fuclosyltransferase1 (*O*-fut1) and Fringe, respectively. After binding the Notch to the ligands (Delta or Serrate, shown in pink), two proteolytic cleavages (S2 and S3 cleavages) are induced. The S2 cleavage is catalyzed by Kuzbanian/ADAM10, consequently removing the Notch extracellular domain. The S3 cleavage is catalyzed by γ-secretase, which occurs in the transmembrane domain of Notch and releases the Notch Intracellular Domain (NICD) from the cell membrane. Consequently, the NICD translocates to the nucleus where it forms a complex with the Mastermind (Mam) and Suppressor of Hairless [Su(H)] and activates the transcription of various target genes. (b) Notch signaling is involved in lateral inhibition and its failure results in a neurogenic phenotype. In *Drosophila* neuroectoderm, a neuroblast (orange) is sorted out from cells composing a proneural cluster (green) through lateral inhibition controlled by Notch signaling. Initially, proneural cluster cells have equivalent potential to choose fate of the neuroblast (left panel). However, in the wild type (upper), once one cell in a proneural cluster starts to acquire the neuroblast's fate, it begins to express *Delta* (*Dl*) which activates the Notch signaling in the adjacent cells (middle). Consequently, Notch signaling prevents adjacent cells from choosing their neuroblast fates, making them epidermoblasts (blue) (left). In the *Notch* mutant (lower), Delta (Dl) is unable to activate Notch signaling in the adjacent cells, which consequently fails to suppress the neuroblast fate (middle). Therefore, all adjacent cells differentiate into neuroblasts (orange) (right).

The *Drosophila* Notch receptor is a transmembrane protein with 36 epidermal growth factor-like repeats in its extracellular domain [32] (Figure 2a). During the Notch protein’s maturation, its extracellular domain is cleaved by the Furin protease (S1 cleavage) within the Golgi apparatus [33–35]. The S1-cleaved Notch protein is then reassembled and moved to the cell’s surface [33–35]. At the surface, the Notch binds to its transmembrane ligand, Delta or Serrate, whose endocytosis induces a mechanical pulling force acting on the Notch’s extracellular domain [36,37]. The pulling force induces a conformational change in the Notch’s extracellular domain, thereby making the protein susceptible to the second cleavage by Kuzbanian/ADAM10 (S2 cleavage). This leads to the extracellular domain’s removal [38,39]. Subsequently, the membrane-tethered form of the Notch intracellular domain (Notch Extracellular Truncation, NEXT) is cleaved within its transmembrane domain by γ-secretase (S3 cleavage), whereby the Notch Intracellular Domain (NICD) is released from the plasma membrane [40]. The NICD then translocates to the nucleus and activates the transcription of target genes [41,42].

**Figure 3.**
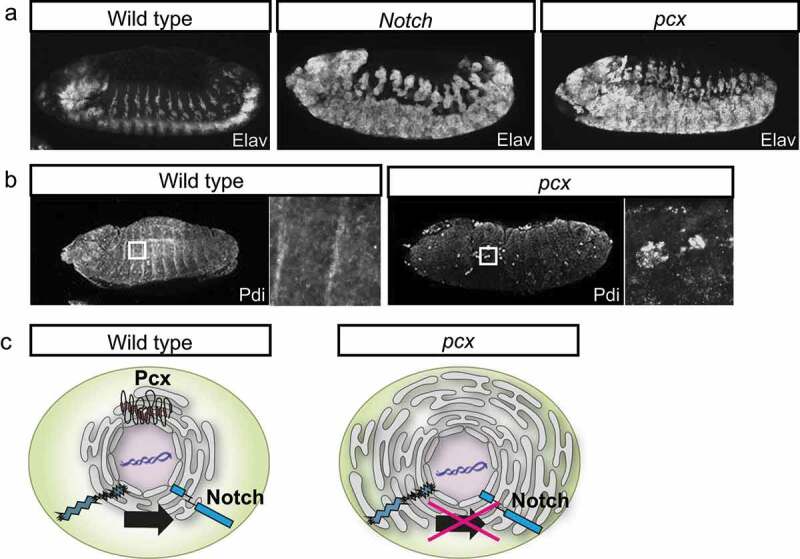
Pcx Promotes the Notch Signaling by Modulating the Endoplasmic Reticulum’s (ER) Physiology. (a) This figure demonstrates the lateral views of the wild-type (left), the *Notch* mutant (middle), and *pcx* maternal/zygotic null embryos (right) at stages 13–14. Neurons are visualized by anti-Elav antibody staining (white). (b) The lateral views of the wild-type (left, wild type) and *pcx* maternal/zygotic null embryos (right, *pcx*) at stages 13–14. The ER is visualized by anti-Pdi antibody staining (white). Right insets show high magnifications of the regions indicated by white boxes in the left panels. (c) Schematics showing the ER structure of cells of the wild type (left, wild type) and the *pcx* maternal/zygotic null (right, *pcx*). The Pcx localizes to the ER where the Notch is properlymatured in wild-type cells (left). However, the ER is enlarged and becomes defective in the absence of Pcx (right).

*Drosophila* Notch signaling plays several important roles in development and homoeostasis. The best-known role of Notch signaling during early development is ‘lateral inhibition’, which prevents the signal-receiving cells in the neuroectoderm from choosing the neuroblast fate [43] (Figure 2b). Therefore, Notch signaling disruption impairs this lateral inhibition and induces neural hyperplasia at the expense of epidermal cells, which is designated as a neurogenic phenotype [43] (Figure 3a). Thus, genes whose mutants show neurogenic phenotypes are referred to as neurogenic genes. Since most of the genes encoding the components of Notch signaling are essential for lateral inhibition, Lehmann et al. (1983) first identified these genes based on the neurogenic phenotypes observed in the corresponding mutants [44].

### The Maternal Members of Genes Composing the Notch Signaling Pathway in Drosophila

Based on the neurogenic phenotypes in embryos, genetic studies have identified five genes as components of Notch signaling: *Notch, big brain* (*bib), mastermind* (*mam), neuralized* (*neur*), and *Delta* (*Dl*) [45]. Since all these mutants show zygotic neurogenic phenotypes, these genes are referred to as zygotic neurogenic genes. However, the zygotic *Notch* mutant’s neurogenic phenotype is weaker than those of the zygotic *mam* mutant, suggesting that *Notch*, but not *mam*, has a maternal contribution [4]. This idea has been confirmed by the observation that homozygous *Notch* or *mam* mutants lacking respective maternal contributions show similar neurogenic phenotypes [4].

Furthermore, various laboratories have identified genes that showed a neurogenic phenotype in embryos lacking maternal and zygotic functions even though their zygotic mutants did not demonstrate a neurogenic phenotype. These genes are designated as ‘maternal neurogenic genes’ and they include *Suppressor of Hairless* [*Su(H)*], *O-fucosyltransferase1* (*O-fut1), Presenilin* (*Psn), anterior pharynx defective 1* (*aph-1), Nicastrin* (*Nct), pecanex* (*pcx), almondex* (*amx), biscotti* (*bisc*), and *amaretto* (*amrt*) [42,46–52]. Thus, their maternally deposited mRNAs and/or translated proteins are maintained after the MZT.

Among them, *Su(H), O-fut1, Psn, aph-1*, and *Nct* are required for Notch signaling maternally, and these genes also function zygotically at later embryonic, larval, pupal and adult stages (maternal and zygotic class), showing that they encode its general components. Since these genes have zygotic functions in Notch signaling, the zygotic mutant of *Su(H)*, for example, shows phenotypes that are found in the zygotic class of neurogenic genes, such as *Notch, Dl*, and *fringe*. At the late embryonic stage of the *Su(H)* zygotic mutant, the proventriculus does not properly form, as in *Notch* zygotic mutant embryos [53]. This may be due to the depletion of the maternal mRNA of *Su*(*H*) by late embryogenesis. The analyses of the maternal and zygotic class neurogenic genes were crucial for our understanding of the signal transduction pathway of Notch signaling. Notably, the basic signal transduction cascade in the Notch signaling pathway was revealed via studies on these genes. On the other hand, *pcx, amx, bisc*, and *amrt* seem to be purely maternally required (purely maternal class) because their zygotic mutants do not show phenotypes related to the ablation of Notch signaling even at late stages of development [50–52]. This may suggest that the genes of the purely maternal class are specifically required for embryonic Notch signaling. However, it is difficult to exclude their roles in post-embryonic Notch signaling because their maternal products, likely proteins, may persist beyond the MZT and potentially until adulthood. This could compensate for the absence of zygotic gene functions in post-embryonic Notch signaling.

Our group has contributed to the analyses of several maternal neurogenic genes, including *O-fut1, pcx*, and *amx* [46,54–57]. Important progress has been made in the understanding of *pcx* and *amx* functions, and the roles of these two maternal neurogenic genes in Notch signaling are discussed below.

### pecanex

*Drosophila pecanex* (*pcx*) was identified as a mutant fly line with recessive female sterility [50]. Namely, homozygous *pcx* females survive until adulthood and oviposit fertilized eggs, whereas embryos deposited from them stop their development and demonstrate neural hyperplasia (Figure 3a). Embryos that are homozygous for *pcx* and lack maternal *pcx* show neural hyperplasia reminiscent of a neurogenic phenotype found in *Notch* mutant embryos (Figure 3a). Furthermore, the maternal neurogenic phenotype of *pcx* was paternally rescued [55,58]. Therefore, it was proposed that *pcx* is a component of the Notch signaling pathway. However, homozygous *pcx* females, with their maternal contribution, do not show detectable developmental defects. Moreover, the contribution of *pcx* to any context of Notch signaling other than embryonic neurogenesis has not been observed [55]. Hence, it is not clear whether *pcx* plays any role in postembryonic Notch signaling. *pcx* encodes a putative sixteen-pass transmembrane protein composed of 3,413 or 3,417 amino acids and is conserved from *Drosophila* to humans [58,59]. In mammals, four tissue-specifically expressed *pcx* paralogs (Pcnx1, Pcmx2, Pcnx3, and Pcnx4) have been identified, although their roles in Notch signaling remain unknown [60].

To deduce the step where *pcx* functions in the Notch signaling cascade, epistasis analyses between *pcx* and various mutant forms of *Notch* have been conducted [55]. The neurogenic phenotypes of the *pcx* maternal and zygotic null are suppressed by the overexpression of NEXT or a nuclear form of the activated Notch (NICD) [55]. These results suggest that *pcx* may act upstream of the S2 and S3 cleavages of Notch. In addition to the epistasis analysis, the cell biological functions of *pcx* have been studied. Embryos homozygous for *pcx* and lacking their maternal contributions show Endoplasmic Reticulum (ER) enlargement [55] (Figure 3b). However, *Notch* mutant embryos do not demonstrate such ER defects, suggesting that the defects associated with the absence of *pcx* may lie upstream of the defects in Notch signaling [55]. This idea is also supplemented by a finding suggesting that *pcx* is an ER-resident protein [55]. Thus, *pcx* has been speculated to play a crucial role in the ER that is essential for normal Notch signaling activation (Figure 3c).

To analyse a potential function of *pcx* in the ER, ER physiology was modulated in the *pcx* heterozygote lacking its maternal contribution (*pcx* maternal/zygotic null). Unfolded Protein Response (UPR) is a cellular response against ER stress induced by accumulating unfolded proteins in the ER [61]. UPR suppresses the cell’s stress levels by reducing the unfolded proteins [61]. The Xbp1 pathway is a major UPR cascade that activates the transcription of genes, encoding chaperone proteins that facilitate protein folding [62,63]. Upregulating the Xbp1 pathway by overexpressing an activated form of *Xbp1* in *pcx* homozygous embryos lacking their maternal contribution rescues their neurogenic phenotype. This observation suggests that the downstream events of the Xbp1 pathway may suppress the ER’s physiological defect. These events include protein synthesis attenuation, enhancement of misfolded ER protein degradation, and induction of genes encoding various chaperones. However, at this point, the way the Xbp1 pathway compensates for the ER defects caused by the absence of the *pcx* function is still unclear.

To identify genes cooperatively functioning with *pcx*, a genetic screen was conducted, which involved a cold-sensitive lethality of the *pcx* maternal/zygotic null [56]. Various gene components of Notch signaling, such as *Delta, bib*, and *neur*, were identified as enhancers and suppressors of lethality, suggesting that the screen fulfilled its purpose. As a dominant suppressor, *N-ethylmaleimide-sensitive factor 2* (*Nsf2*), encoding a key regulator of vesicular fusion, was isolated from the screen [56]. *NSF* is known to control the cohesion of ER [64,65]. Considering that the loss of the *pcx* function induces ER enlargement, *Nsf2* and *pcx* may be antagonistic in the homoeostasis of ER morphology [56].

### almondex

**Figure 4.**
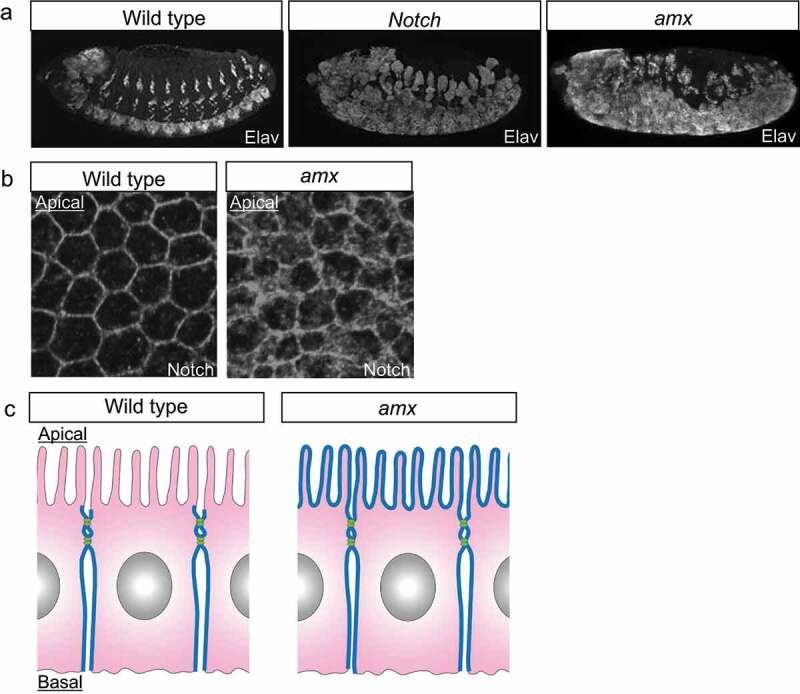
Amx Promotes Notch Signaling by Regulating the Notch’s Subcellular Localization. (a) The lateral views of the wild-type (left, wild type), the *Notch* mutant (middle, *Notch*), and *amx* maternal/zygotic null embryos (right, *amx*) at stages 13–14. Neurons are visualized by anti-Elav antibody staining (white). (b) The distribution of Notch (white) at the apical regions of epithelial cells in the wild type (wild type) and *amx* maternal/zygotic null embryos at stage 5. (c) Schematics of the Notch (blue) localization in the epithelium of the wild type (left, wild type) and the *amx* maternal/zygotic null (right, *amx*).

In *Drosophila, amx* was identified as a maternal neurogenic gene because fertilized eggs obtained from *amx* homozygous mothers showed strong neurogenic phenotypes [44,45,51,66] (Figure 4a). Therefore, *amx* is considered a component of the Notch signaling pathway. In the *Drosophila* genome, three paralogs of *amx* exist, namely *biscotti* (*bisc), amx*, and *amaretto* (*amrt*) [52]. A study revealed that all three are maternal neurogenic genes, implying that each of them plays maternal roles that are essential for Notch signaling [52]. However, triple homozygotes of *bisc, amx*, and *amrt* are viable due to the maternal contribution of the corresponding proteins. These mutants do not show detectable phenotypes in development until adulthood, demonstrating that *amx* paralogs have no zygotic role in the postembryonic development in *Drosophila* [52]. The *amx* family genes are evolutionarily conserved, and their mammalian orthologs are designated as the TM2 domain (TM2D) containing protein genes [67,68]. *bisc, amx*, and *amrt* correspond to mammalian orthologs: *TM2D1, TM2D2*, and *TM2D3*, respectively [52]. These *amx* family genes encode predicted double-pass transmembrane proteins with a conserved DRF motif and a TM2 domain [68]. Their structure and functions are conserved between *Drosophila* and mammalian orthologs because *TM2D3* can rescue the neurogenic phenotype of *Drosophila amx* mutant embryos [69].

The *Drosophila* model led to the development of an understanding of the *amx* gene’s functions. Genetic epistasis between *amx* and *Notch* provided important insights for understanding where *amx* is required in the Notch signaling cascade [68]. The overexpression of a full-length Notch or NEXT fails to suppress the neurogenic phenotype in embryos that are homozygous for *amx* and devoid of the corresponding maternal contribution. On the contrary, the NICD’s overexpression suppresses the phenotypes [68]. These results suggest that *amx* may function at the level of the Notch S3 cleaves. More recently, another epistasis analysis was conducted between various forms of *Notch* and a mutant *amx*, encoding a derivative of *amx* with an N-terminus truncation (AMX^ECD^), a potent inhibitor of Notch signaling [52]. This study revealed that overexpression of AMX^ECD^ suppresses the accelerated Notch signaling activity by NEXT or a ligand-dependent activated Notch, but does not suppress NICD [52]. Thus, taken together, these results suggest that *amx* acts after the production of NEXT and before the production of NICD in the Notch signaling cascade.

Researchers have also started to understand the cell biological functions of *amx*. In embryos homozygous for *amx* (*amx* maternal/zygotic nulls), Notch is abnormally accumulated in the neuroectoderm even though such defective localization is restored with the progression of embryonic development [57] (Figure 4b). This observation suggests that *amx* may regulate the intracellular trafficking of Notch in a stage-specific manner [57] (Figure 4c). This idea is consistent with the results of epistasis analysis demonstrating that *amx* acts before the NICD production [52,68]. In addition to such functions of *amx* associated with the roles of the Notch signaling in embryonic development, studies have revealed the physiological roles of *amx* in adult flies [52]. The lifespan of adult flies homozygous for *amx* is shorter than that of wild types [52]. Additionally, an age-dependent decline in neural functions was observed in these adults [52]. Although it is currently unknown whether these defects are associated with reduced Notch signaling, these results demonstrated that *amx* plays a crucial role in adult physiology and ageing.

The involvement of *amx* family genes in human diseases has also been reported. A rare missense variant (P155L) in *TM2D3* is significantly associated with an increased risk of developing late-onset Alzheimer’s disease and an earlier age of onset [69]. This missense mutation behaves as a loss-of-function mutant of *TM2D3* in *Drosophila* [69]. Considering that *TM2D3* affects Notch signaling and Alzheimer’s disease, the γ-secretase that plays crucial roles in both events may be a target of *TM2D3*. Accordingly, it has been proposed that *TM2D3* may participate in the intramembrane cleavage of Notch and APP by γ-secretase. Additionally, the P155L variant may influence the production of the β-amyloid peptide, which is similar to the pathogenic mutant forms of APP, Presenilin-1, and Presenilin-2 [69]. Thus, it was proposed that the Amx-family proteins are likely involved in γ-secretase functions and the production of the Aβ-42 peptide [67,68]. This idea is further supported by a previous finding that mammalian TM2D proteins bind the Aβ-42 peptide, which may help in the clearance of amyloid plaques by phagocytic glia cells [67]. Thus, the functional relevance between the *amx* family genes and the γ-secretase seems to be a promising subject for future research.

### Maternal Notch Signaling in Other Animals

We will discuss the maternal effect of Notch signaling in various organisms. In invertebrate models, studies have revealed the essential roles of maternal genes encoding the components of the Notch signaling in embryogenesis. In *Caenorhabditis elegans* (*C. elegans*), there are two homologs of Notch, namely the *abnormal cell lineage-12* (*lin-12*) and *abnormal germ line proliferation-1* (*glp-1*) [70,71]. Mutants of *glp-1* were rediscovered based on their postembryonic sterility [72]. Subsequently, it was found that *glp-1* shows a recessive maternal effect of lethality because *glp-1* homozygous mothers produce only abnormal progenies regardless of the genotypes of embryos [73]. In the absence of the maternal *glp-1*, the AB-derived blastomeres, which normally produce pharyngeal cells, produce neurons instead [73]. In *C. elegans*, orthologs of *pcx* and *amx* have been reported [74,75]. However, phenotypes associated with their loss of functions have not. In sea urchins, a model invertebrate belonging to deuterostomia, *Notch* and *Delta* orthologs are expressed during early embryogenesis. Additionally, they play maternal roles [76]. Maternal genes of sea urchins maintain high expression until the two-cell stage, which decreases after the expression of zygotic genes [76]. Using a Morpholino, *Notch* and *fringe* proteins – which encode *O*-fucose-specific β-1,3 *N*-acetylglucosaminyltransferase – were knocked down at the two-cell stage [77]. These embryos fail to gastrulate, indicating that maternal Notch signaling requires glycosylation of Notch EGF-repeats by Fringe in sea urchins [77].

In contrast to invertebrates, the maternal effect’s contribution to Notch signaling in vertebrates is still ambiguous. Mouse embryos that are homozygous for *O-fut1* and lack maternal contributions, in which maternal Notch signaling should be abolished, normally developed until E8 [78]. In mice, the expression levels of maternal and zygotic genes are reversed at the two-cell stage (E1.5). Embryos lacking the maternal effect cannot survive the cleavage-stage corresponding to the first three cell divisions before forming a mature morula [79] (Figure 2). Therefore, Notch signaling is not essential for morphogenesis before gastrulation, indicating that the maternal effect is not involved in mammalian Notch signaling. However, the definition of maternal genes in mammals has been expanded. This is due to the discovery of new maternal genes that play an essential role in embryonic development after the two-cell stage [80]. Additionally, it has been reported that the administration of an inhibitor of Notch signaling to mouse embryos at the two-cell stage downregulates *TLE4*, an early naïve-pluripotency marker [81]. Therefore, further studies are required to determine whether maternal functions of genes play essential roles in Notch signaling.

## Conclusions

*Drosophila* embryos complete cellularization and begin cell-cell interactions at stage 5. Their neuroblasts begin differentiation in the neuroectoderm at stage 8 [29]. Therefore, lateral inhibition through Notch signaling should occur during stages 5–8. Genetic screens based on the zygotic neurogenic phenotype occasionally failed to isolate maternal neurogenic genes. This is because many genes composing the Notch signaling pathway were found to have maternal contributions [42,46–52]. Most newly identified genes in Notch signaling are maternal neurogenic genes. Thus, further studies on maternal genes will be useful for identifying novel components of Notch signaling and other cell signaling pathways. This idea applies to organisms having large quantities of maternal mRNAs and proteins.

Most *Drosophila* maternal neurogenic mutants are also recessive zygotic lethal mutations. Thus, these maternal neurogenic genes also play zygotic roles that are essential for embryonic and postembryonic development. However, homozygotes of *pcx* or *amx* family genes with their maternal contributions do not show postembryonic defects in development and are viable [50,66]. These phenomena can be explained if the maternal contributions of these genes persist until adulthood. However, alternatively, these genes may have specific roles only in the Notch signaling of the embryonic stage. In the latter case, we must consider another layer of complexity in Notch signaling regulation, such as stage-specific regulatory factors, which may further extend our understanding of this signaling pathway.

The MZT has been an important subject in developmental biology. Many maternal genes play essential roles in cell signaling. Accordingly, understanding the balance between the maternal and zygotic functions of genes involved in cell signaling and their regulation during the MZT is of the utmost importance.

## Data Availability

Data sharing does not apply to this article as no new data were created or analyzed in this study.

## References

[1] Boycott AE, Diver C. On the inheritance of sinistrality in Limnæa peregra. Proc R Soc Lond B Biol Sci. 1923;95(666):207–213.

[2] Sturtevant AH. Inheritance of Direction of Coiling in Limnaea. Science. 1923;58(1501):269–270. 17837785 10.1126/science.58.1501.269

[3] Frohnhӧfer HG, Nfisslein-Volhard C. Organization, of anterior pattern in the Drosophila embryo by the maternal gene bicoid. Nature. 1986;324(6093):120–125.

[4] Jiménez F, Campos-Ortega JA. Maternal effects of zygotic mutants affecting early neurogenesis in Drosophila. Wilhelm Roux’s Arch. 1982;191(3):191–201 10.1007/BF00848335 28305383

[5] Weeks DL, Melton DA. A maternal mRNA localized to the vegetal hemisphere in Xenopus eggs codes for a growth factor related to TGF-beta. Cell. 1987;51(5):861–867. 3479264 10.1016/0092-8674(87)90109-7

[6] Morisato D, Anderson KV. Signaling pathways that establish the dorsal-ventral pattern of the drosophila embryo. Annu Rev Genet. 1995;29(1):371–399. 8825480 10.1146/annurev.ge.29.120195.002103

[7] Kelly C, Chin AJ, Leatherman JL, et al. Maternally controlled β-catenin-mediated signaling is required for organizer formation in the zebrafish. Development. 2000;127(18):3899–3911. 10952888 10.1242/dev.127.18.3899

[8] Tao Q, Yokota C, Puck H, et al. Maternal wnt11 activates the canonical wnt signaling pathway required for axis formation in Xenopus embryos. Cell. 2005;120(6):857–871. 15797385 10.1016/j.cell.2005.01.013

[9] Tadros W, Goldman AL, Babak T, et al. SMAUG is a major regulator of maternal mRNA destabilization in Drosophila and its translation is activated by the PAN GU kinase. Dev Cell. 2007;12(1):143–155. 17199047 10.1016/j.devcel.2006.10.005

[10] Lecuyer E, Yoshida H, Parthasarathy N, et al. Global analysis of mRNA localization reveals a prominent role in organizing cellular architecture and function. Cell. 2007;131(1):174–187. 17923096 10.1016/j.cell.2007.08.003

[11] De Renzis S, Elemento O, Tavazoie S, et al. Unmasking activation of the zygotic genome using chromosomal deletions in the Drosophila embryo. PLoS Biol. 2007;5(5):e117. 17456005 10.1371/journal.pbio.0050117 PMC1854917

[12] Tadros W, Lipshitz HD. The maternal-to-zygotic transition: a play in two acts. Development. 2009;136(18):3033–3042. 19700615 10.1242/dev.033183

[13] Seydoux G, Fire A. Soma-germline asymmetry in the distributions of embryonic RNAs in Caenorhabditis elegans. Development. 1994;120(10):2823–2834. 7607073 10.1242/dev.120.10.2823

[14] Bashirullah A, Halsell SR, Cooperstock RL, et al. Joint action of two RNA degradation pathways controls the timing of maternal transcript elimination at the midblastula transition in Drosophila melanogaster. EMBO J. 1999;18(9):2610–2620. 10228172 10.1093/emboj/18.9.2610 PMC1171340

[15] Hamatani T, Carter MG, Sharov AA, et al. Dynamics of global gene expression changes during mouse preimplantation development. Dev Cell. 2004;6(1):117–131. 14723852 10.1016/s1534-5807(03)00373-3

[16] Gildor T, Ben-Tabou de-Leon S. Comparative study of regulatory circuits in two sea urchin species reveals tight control of timing and high conservation of expression dynamics. PLoS Genet. 2015;11(7):e1005435. 26230518 10.1371/journal.pgen.1005435 PMC4521883

[17] Foe VE, Alberts BM. Studies of nuclear and cytoplasmic behaviour during the five mitotic cycles that precede gastrulation in Drosophila embryogenesis. J Cell Sci. 1983;61(1):31–70. 6411748 10.1242/jcs.61.1.31

[18] Lott SE, Villalta JE, Schroth GP, et al. Noncanonical compensation of zygotic X transcription in early Drosophila melanogaster development revealed through single-embryo RNA-seq. PLoS Biol. 2011;9(2):e1000590. 21346796 10.1371/journal.pbio.1000590 PMC3035605

[19] Bushati N, Stark A, Brennecke J, et al. Temporal reciprocity of miRNAs and their targets during the maternal-to-zygotic transition in Drosophila. Curr Biol. 2008;18(7):501–506. 18394895 10.1016/j.cub.2008.02.081

[20] Thomsen S, Anders S, Janga SC, et al. Genome-wide analysis of mRNA decay patterns during early Drosophila development. Genome Biol. 2010;11(9):R93. 20858238 10.1186/gb-2010-11-9-r93 PMC2965385

[21] Siddiqui NU, Li X, Luo H, et al. Genome-wide analysis of the maternal-to-zygotic transition in Drosophila primordial germ cells. Genome Biol. 2012;13(2):R11. 22348290 10.1186/gb-2012-13-2-r11 PMC3334568

[22] Cao WX, Kabelitz S, Gupta M, et al. Precise temporal regulation of post-transcriptional repressors is required for an orderly drosophila maternal-to-zygotic transition. Cell Rep. 2020;31(12):107783. 32579915 10.1016/j.celrep.2020.107783 PMC7372737

[23] Liang HL, Nien CY, Liu HY, et al. The zinc-finger protein Zelda is a key activator of the early zygotic genome in Drosophila. Nature. 2008;456(7220):400–403. 18931655 10.1038/nature07388 PMC2597674

[24] Harrison MM, Botchan MR, Cline TW. Grainyhead and Zelda compete for binding to the promoters of the earliest-expressed Drosophila genes. Dev Biol. 2010;345(2):248–255. 20599892 10.1016/j.ydbio.2010.06.026 PMC2927720

[25] Nien CY, Liang HL, Butcher S, et al. Temporal coordination of gene networks by Zelda in the early Drosophila embryo. PLoS Genet. 2011;7(10):e1002339. 22028675 10.1371/journal.pgen.1002339 PMC3197689

[26] ten Bosch JR, Benavides JA, Cline TW. The TAGteam DNA motif controls the timing of Drosophila pre-blastoderm transcription. Development. 2006;133(10):1967–1977. 16624855 10.1242/dev.02373

[27] Sun Y, Nien CY, Chen K, et al. Zelda overcomes the high intrinsic nucleosome barrier at enhancers during Drosophila zygotic genome activation. Genome Res. 2015;25(11):1703–1714. 26335633 10.1101/gr.192542.115 PMC4617966

[28] Schulz KN, Bondra ER, Moshe A, et al. Zelda is differentially required for chromatin accessibility, transcription-factor binding and gene expression in the early Drosophila embryo. Genome Res. 2015;25(11):1715–1726. 26335634 10.1101/gr.192682.115 PMC4617967

[29] Hartenstein V, Campos-Ortega JA. Early neurogenesis in wild-type Drosophila melanogaster. Wilehm Roux Arch Dev Biol. 1984;193(5):308–325 28305340 10.1007/BF00848159

[30] Menne TV, Klambt C. The formation of commissures in the Drosophila CNS depends on the midline cells and on the Notch gene. Development. 1994;120(1):123–133. 8119121 10.1242/dev.120.1.123

[31] Martin-Bermudo MD, Carmena A, Jiménez F. Neurogenic genes control gene expression at the transcriptional level in early neurogenesis and in mesectoderm specification. Development. 1995;121(1):219–224. 7867503 10.1242/dev.121.1.219

[32] Wharton KA, Johansen KM, Xu T, et al. Nucleotide sequence from the neurogenic locus notch implies a gene product that shares homology with proteins containing EGF-like repeats. Cell. 1985;43(3 Pt 2):567–581. 3935325 10.1016/0092-8674(85)90229-6

[33] Logeat F, Bessia C, Brou C, et al. The Notch1 receptor is cleaved constitutively by a furin-like convertase. Proc Natl Acad Sci USA. 1998;95(14):8108–8112. 9653148 10.1073/pnas.95.14.8108 PMC20937

[34] Kidd S, Lieber T. Furin cleavage is not a requirement for Drosophila Notch function. Mech Dev. 2002;115(1–2):41–51. 12049766 10.1016/s0925-4773(02)00120-x

[35] Lake RJ, Grimm LM, Veraksa A, et al. In vivo analysis of the Notch receptor S1 cleavage. PLoS ONE. 2009;4(8):e6728. 19701455 10.1371/journal.pone.0006728 PMC2726433

[36] Wang W, Struhl G. Distinct roles for Mind bomb, Neuralized and Epsin in mediating DSL endocytosis and signaling in Drosophila. Development. 2005;132(12):2883–2894. 15930117 10.1242/dev.01860

[37] Meloty-Kapella L, Shergill B, Kuon J, et al. Notch ligand endocytosis generates mechanical pulling force dependent on dynamin, epsins, and actin. Dev Cell. 2012;22(6):1299–1312. 22658936 10.1016/j.devcel.2012.04.005 PMC3400432

[38] Kopan R, Goate A. A common enzyme connects notch signaling and Alzheimer’s disease. Genes Dev. 2000;14(22):2799–2806. 11090127 10.1101/gad.836900

[39] Stephenson NL, Avis JM. Direct observation of proteolytic cleavage at the S2 site upon forced unfolding of the Notch negative regulatory region. Proc Natl Acad Sci USA. 2012;109(41):E2757–E2765. 23011796 10.1073/pnas.1205788109 PMC3478610

[40] Mumm JS, Kopan R. Notch signaling: from the outside in. Dev Biol. 2000;228(2):151–165. 11112321 10.1006/dbio.2000.9960

[41] Struhl G, Fitzgerald K, Greenwald I. Intrinsic activity of the Lin12 and Notch intracellular domains in vivo. Cell. 1993;74(2):331–345. 8343960 10.1016/0092-8674(93)90424-o

[42] Lecourtois M, Schweisguth F. The neurogenic suppressor of hairless DNA-binding protein mediates the transcriptional activation of the enhancer of split complex genes triggered by Notch signaling. Genes Dev. 1995;9(21):2598–2608. 7590238 10.1101/gad.9.21.2598

[43] Simpson P. Lateral inhibition and the development of the sensory bristles of the adult peripheral nervous system of Drosophila. Development. 1990;109(3):509–519. 2205467 10.1242/dev.109.3.509

[44] Lehmann R, Jiménez F, Dietrich U, et al. On the phenotype and development of mutants of early neurogenesis in Drosophila melanogaster. Rouxs Arch Dev Biol. 1983;192(2):62–74 10.1007/BF00848482 28305500

[45] Lehmann R, Dietrich U, Jiménez F, et al. Mutations of early neurogenesis in Drosophila. Rouxs Arch Dev Biol. 1981;190(4):226–229 10.1007/BF00848307 28305572

[46] Sasamura T, Sasaki N, Miyashita F, et al. neurotic, a novel maternal neurogenic gene, encodes an O-fucosyltransferase that is essential for Notch-Delta interactions. Development. 2003;130(20):4785–4795. 12917292 10.1242/dev.00679

[47] Ye Y, Lukinova N, Fortini ME. Neurogenic phenotypes and altered Notch processing in Drosophila Presenilin mutants. Nature. 1999;398(6727):525–529. 10206647 10.1038/19096

[48] Hu Y, Fortini ME. Different cofactor activities in gamma-secretase assembly: evidence for a nicastrin-Aph-1 subcomplex. J Cell Biol. 2003;161(4):685–690. 12771124 10.1083/jcb.200304014 PMC2199374

[49] Lopez-Schier H, St Johnston D. Drosophila nicastrin is essential for the intramembranous cleavage of Notch. Dev Cell. 2002;2(1):79–89. 11782316 10.1016/s1534-5807(01)00109-5

[50] Perrimon N, Engstrom L, Mahowald AP. Developmental genetics of the 2E-F region of the Drosophila X chromosome: a region rich in “developmentally important” genes. Genetics. 1984;108(3):559–572. 6437900 10.1093/genetics/108.3.559 PMC1202425

[51] Michellod MA, Forquignon F, Santamaria P, et al. Differential requirements for the neurogenic gene almondex during Drosophila melanogaster development. Genesis. 2003;37(3):113–122. 14595834 10.1002/gene.10233

[52] Salazar JL, Yang SA, Lin YQ, et al. TM2D genes regulate Notch signaling and neuronal function in Drosophila. PLoS Genet. 2021;17(12):e1009962. 34905536 10.1371/journal.pgen.1009962 PMC8714088

[53] Fuss B, Josten F, Feix M, et al. Cell movements controlled by the Notch signalling cascade during foregut development in Drosophila. Development. 2004;131(7):1587–1595. 14998929 10.1242/dev.01057

[54] Sasamura T, Ishikawa HO, Sasaki N, et al. The O-fucosyltransferase O-fut1 is an extracellular component that is essential for the constitutive endocytic trafficking of Notch in Drosophila. Development. 2007;134(7):1347–1356. 17329366 10.1242/dev.02811

[55] Yamakawa T, Yamada K, Sasamura T, et al. Deficient Notch signaling associated with neurogenic pecanex is compensated for by the unfolded protein response in Drosophila. Development. 2012;139(3):558–567. 22190636 10.1242/dev.073858

[56] Yamakawa T, Atsumi Y, Kubo S, et al. Insight into notch signaling steps that involve pecanex from dominant-modifier screens in drosophila. Genetics. 2018;209(4):1099–1119. 29853475 10.1534/genetics.118.300935 PMC6063225

[57] Das P, Salazar JL, Li-Kroeger D, et al. Maternal almondex, a neurogenic gene, is required for proper subcellular Notch distribution in early Drosophila embryogenesis. Dev Growth Differ. 2020;62(1):80–93. 31782145 10.1111/dgd.12639

[58] LaBonne SG, Sunitha I, Mahowald AP. Molecular genetics of pecanex, a maternal-effect neurogenic locus of Drosophila melanogaster that potentially encodes a large transmembrane protein. Dev Biol. 1989;136(1):1–16. 2478400 10.1016/0012-1606(89)90127-9

[59] Roux AF, Rommens JM, Read L, et al. Physical and transcription map in the region 14q24.3: identification of six novel transcripts. Genomics. 1997;43(2):130–140. 9244429 10.1006/geno.1997.4786

[60] Gilbert TL, Haldeman BA, Mulvihill E, et al. A mammalian homologue of a transcript from the Drosophila pecanex locus. J Neurogenet. 1992;8(3):181–187. 1460533 10.3109/01677069209083447

[61] Kaufman RJ. Stress signaling from the lumen of the endoplasmic reticulum: coordination of gene transcriptional and translational controls. Genes Dev. 1999;13(10):1211–1233. 10346810 10.1101/gad.13.10.1211

[62] Lee AH, Iwakoshi NN, Glimcher LH. XBP-1 regulates a subset of endoplasmic reticulum resident chaperone genes in the unfolded protein response. Mol Cell Biol. 2003;23(21):7448–7459. 14559994 10.1128/MCB.23.21.7448-7459.2003 PMC207643

[63] Yoshida H, Matsui T, Hosokawa N, et al. A time-dependent phase shift in the mammalian unfolded protein response. Dev Cell. 2003;4(2):265–271. 12586069 10.1016/s1534-5807(03)00022-4

[64] Wilson DW, Wilcox CA, Flynn GC, et al. A fusion protein required for vesicle-mediated transport in both mammalian cells and yeast. Nature. 1989;339(6223):355–359. 2657434 10.1038/339355a0

[65] Söllner T, Whiteheart SW, Brunner M, et al. SNAP receptors implicated in vesicle targeting and fusion. Nature. 1993;362(6418):318–324. 8455717 10.1038/362318a0

[66] Shannon MP. Characterization of the female-sterile mutant almondex of Drosophila melanogaster. Genetica. 1972;43(2):244–256. 4625164 10.1007/BF00123632

[67] Kajkowski EM, Lo CF, Ning X, et al. beta -Amyloid peptide-induced apoptosis regulated by a novel protein containing a g protein activation module. J Biol Chem. 2001;276(22):18748–18756. 11278849 10.1074/jbc.M011161200

[68] Michellod MA, Randsholt NB. Implication of the Drosophila beta-amyloid peptide binding-like protein AMX in Notch signaling during early neurogenesis. Brain Res Bull. 2008;75(2–4):305–309. 18331889 10.1016/j.brainresbull.2007.10.060

[69] Jakobsdottir J, van der Lee SJ, Bis JC, et al. Rare functional variant in TM2D3 is associated with Late-Onset Alzheimer’s Disease. PLoS Genet. 2016;12(10):e1006327. 27764101 10.1371/journal.pgen.1006327 PMC5072721

[70] Yochem J, Weston K, Greenwald I. The Caenorhabditis elegans lin-12 gene encodes a transmembrane protein with overall similarity to Drosophila Notch. Nature. 1988;335(6190):547–550. 3419531 10.1038/335547a0

[71] Yochem J, Greenwald I. glp-1 and lin-12, genes implicated in distinct cell-cell interactions in C. elegans, encode similar transmembrane proteins. Cell. 1989;58(3):553–563. 2758466 10.1016/0092-8674(89)90436-4

[72] Austin J, Kimble J. glp-1 is required in the germ line for regulation of the decision between mitosis and meiosis in C. elegans. Cell. 1987;51(4):589–599. 3677168 10.1016/0092-8674(87)90128-0

[73] Priess JR, Schnabel H, Schnabel R. The glp-1 locus and cellular interactions in early C. elegans embryos. Cell. 1987;51(4):601–611. 3677169 10.1016/0092-8674(87)90129-2

[74] Shaye DD, Greenwald I. OrthoList: a compendium of C. elegans genes with human orthologs. PLoS One. 2011;6(5):e20085. 21647448 10.1371/journal.pone.0020085 PMC3102077

[75] Li R, Ren X, Ding Q, et al. Direct full-length RNA sequencing reveals unexpected transcriptome complexity during Caenorhabditis elegans development. Genome Res. 2020;30(2):287–298. 32024662 10.1101/gr.251512.119 PMC7050527

[76] Walton KD, Croce JC, Glenn TD, et al. Genomics and expression profiles of the Hedgehog and Notch signaling pathways in sea urchin development. Dev Biol. 2006;300(1):153–164. 17067570 10.1016/j.ydbio.2006.08.064 PMC1880897

[77] Peterson RE, McClay DR. A Fringe-modified Notch signal affects specification of mesoderm and endoderm in the sea urchin embryo. Dev Biol. 2005;282(1):126–137. 15936334 10.1016/j.ydbio.2005.02.033

[78] Shi S, Stahl M, Lu L, et al. Canonical notch signaling is dispensable for early cell fate specifications in mammals. Mol Cell Biol. 2005;25(21):9503–9508. 16227600 10.1128/MCB.25.21.9503-9508.2005 PMC1265842

[79] Li L, Zheng P, Dean J. Maternal control of early mouse development. Development. 2010;137(6):859–870. 20179092 10.1242/dev.039487 PMC2834456

[80] Zheng P, Dean J. Role of Filia, a maternal effect gene, in maintaining euploidy during cleavage-stage mouse embryogenesis. Proc Natl Acad Sci U S A. 2009;106(18):7473–7478. 19376971 10.1073/pnas.0900519106 PMC2678599

[81] Menchero S, Rollan I, Lopez-Izquierdo A, et al. Transitions in cell potency during early mouse development are driven by Notch. eLife. 2019;8:e42930. 30958266 10.7554/eLife.42930 PMC6486152

